# Trained Immunity in Bladder ILC3s Enhances Mucosal Defense Against Recurrent Urinary Tract Infections

**DOI:** 10.3390/biomedicines14010078

**Published:** 2025-12-30

**Authors:** Qiaoqiao Pei, Jiaqi Liu, Ziwen Tang, Jiaqing Tan, Xu Han, Xinrong Hu, Zhou Liang, Feng Li, Changjian Zhu, Ruoni Lin, Ruilin Zheng, Jiani Shen, Qinghua Liu, Haiping Mao, Kefei Wu, Wei Chen, Yi Zhou

**Affiliations:** 1 Department of Nephrology, The First Affiliated Hospital, Sun Yat-sen University, Guangzhou 510080, China; 2 NHC Key Laboratory of Clinical Nephrology (Sun Yat-sen University) and Guangdong Provincial Key Laboratory of Nephrology, Guangzhou 510080, China; 3 Center of Nephrology, Jieyang People’s Hospital, Jieyang 522000, China

**Keywords:** group 3 innate lymphoid cells, trained immunity, innate immune memory, urinary tract infection, uropathogenic *Escherichia coli*

## Abstract

**Background**: Urinary tract infections (UTIs) rank among the most prevalent infectious diseases globally, with recurrent UTIs (rUTIs) posing substantial therapeutic challenges due to the lack of durable protective immunity. While trained immunity augments innate immune responses, its induction and functional significance in bladder-resident group 3 innate lymphoid cells (ILC3s) remain unknown. This study investigates whether ILC3s develop trained immunity following uropathogenic *Escherichia coli* (UPEC) exposure and how they contribute to mucosal defense against rUTIs. **Methods**: The ILC3 counts were detected in bladder sections from UTI patients and health controls (HC). A recurrent UTI mouse model was established through primary and secondary urethral UPEC inoculation. Bacterial loads in tissues were assessed, and single-cell suspensions were analyzed via flow cytometry. Bladder naïve- and UPEC-trained ILC3s were adoptively transferred, with evaluations of histopathology, epithelial barrier function, inflammation, and antimicrobial peptides. The in vitro ILC3 cell line MNK-3 was detected for IL-17A and IL-22 production following primary and secondary UPEC lysate stimulation. **Results**: We demonstrate that primary UPEC infection triggers ILC3 expansion in both human and murine bladders. Upon secondary challenge, these ILC3s develop trained immunity, characterized by enhanced proliferation, amplified IL-17A and IL-22 production, and improved pathogen clearance. Mechanistically, trained ILC3s reinforce urothelial barrier integrity through upregulation of antimicrobial peptides (Reg3b/Reg3g) and attenuate inflammatory pathology by suppressing pro-inflammatory cytokines (IL-6, TNF-α). **Conclusions**: We uncover an endogenous defense mechanism wherein UPEC primes bladder ILC3s via trained immunity, enabling amplified IL-17A- and IL-22-mediated protection against recurrent infections. These findings establish ILC3-trained immunity as a novel conceptual foundation, providing a basis for developing immunotherapies against rUTIs.

## 1. Introduction

Recurrent urinary tract infections (rUTIs) pose a formidable and escalating clinical challenge, affecting millions globally and significantly impacting patient quality of life despite conventional antibiotic therapies [1,2,3,4]. The high recurrence rate, often exceeding 30% within six months, underscores a critical gap in our understanding of host defense mechanisms and highlights the urgent need for novel therapeutic strategies beyond pathogen eradication [4,5,6,7]. This high recurrence rate presents a fundamental immunological contradiction: the initial infection fails to establish a state of protective immunity that adequately prevents subsequent episodes. This implies that the host’s intrinsic defense mechanisms are either insufficient or fail to form adequate immune memory. If the host immune system could establish effective, durable responses after initial infection, recurrences would be less common. Thus, the very existence of rUTIs provides a compelling rationale for exploring innate immune memory, particularly trained immunity, as a crucial missing link in addressing this conundrum.

The innate immune system serves as the first line of defense against UTIs and plays well-characterized roles in pathogen clearance [8]. More recently, innate lymphoid cells (ILCs) have emerged as crucial mediators of mucosal immunity through cytokine production, reinforcing mucosal barriers, and modulating immune responses and tissue inflammation [9,10,11,12,13,14,15,16,17,18]. Among ILC subgroups, group 3 ILCs (ILC3s) are particularly important for antibacterial defense, producing interleukin-17A (IL-17A) and IL-22 to coordinate mucosal immunity [19,20,21]. While recent work, including that by Huang et al., has established the protective role of ILC3s in acute UPEC infection by demonstrating increased susceptibility to infection upon ILC3 depletion, the precise mechanisms by which these cells contribute to sustained protection against recurrent infections remain largely undefined [22]. Crucially, the capacity of bladder-resident ILC3s to develop “trained immunity”—a form of innate immune memory that confers enhanced, long-lasting protection against subsequent challenges—has not been investigated [23].

Trained immunity enhances innate immune responses to secondary challenges through functional reprogramming, enabling a more robust and efficient response to secondary infections [24,25,26,27,28,29]. While trained immunity has been observed in other ILC subsets and at different mucosal sites, such as intestinal ILC3s following *C. rodentium* infection, its existence and functional significance in bladder-resident ILC3s in the context of recurrent UTIs are entirely unknown [10,11,16,30,31,32]. This represents a critical gap, as understanding such an innate memory mechanism could unlock novel strategies for long-term UTI prevention.

Here, we provide the first evidence that bladder ILC3s acquire trained immunity following UPEC exposure, leading to a significantly enhanced and durable protective response against recurrent UTIs. We demonstrate that primary UPEC infection induces a distinct ‘trained’ phenotype in bladder ILC3s, a phenomenon we term “ILC3-trained immunity”, characterized by augmented proliferative capacity and heightened production of IL-17A and IL-22 upon re-challenge. Crucially, through adoptive transfer experiments, we show that these trained ILC3s (Tr-ILC3s) confer superior protection against recurrent infection compared to their naïve counterparts, effectively reducing bacterial burden, reinforcing urothelial barrier integrity via Reg3b/Reg3g upregulation, and attenuating inflammatory responses. These findings not only unveil a previously unrecognized mechanism of innate immune memory in the urinary tract but also position ILC3-trained immunity as a promising and innovative therapeutic strategy to combat the persistent challenge of recurrent UTIs.

## 2. Materials and Methods

### 2.1. Human Samples

All bladder tissue samples in this study were obtained from patients undergoing biopsy or surgical resection for suspected or confirmed bladder neoplasms. The UTI group comprised tissue samples from patients who were concurrently diagnosed with urinary tract infection, based on medical records indicating the presence of pyuria, bacteriuria (positive urine culture), and relevant symptoms such as dysuria, urinary frequency, urgency, or suprapubic discomfort. The control group consisted of histologically normal-appearing marginal tissues from the resection specimens of patients who had no clinical or laboratory evidence of concurrent UTI. These control tissues were pathologically confirmed to be free of tumor. Exclusion criteria included: concurrent infections at other sites, severe complications (e.g., renal insufficiency, immunodeficiency, diabetes mellitus), pregnant or lactating women, individuals with urinary tract anatomical abnormalities (such as urinary calculi, malformations, or obstructions), and patients unable to comply with treatment or follow-up protocols. Patients over 18 years of age were enrolled in the study, regardless of sex. All participants provided informed consent and this study adhered to the Declaration of Helsinki and was approved by the Ethical Committee of Jieyang People’s Hospital (Jieyang Affiliated Hospital, Sun Yat-sen University) (2025009).

### 2.2. Bacterial Strains

Uropathogenic *Escherichia coli* (*E. coli*) strain CFT073 (ATCC 700928, isolated from an acute pyelonephritis patient’s clinical specimen) was used. To confirm its identity, bacterial genomic DNA was extracted and the 16S ribosomal DNA (rDNA) gene was amplified by PCR using universal primers 27F (5′-AGAGTTTGATCCTGGCTCAG-3′) and 1492R (5′-GGTTACCTTGTTACGACTT-3′). The purified PCR product was sequenced, and the resulting sequence was analyzed using the BLAST algorithm (version 2.17.0) against the NCBI GenBank database. The strain showed the highest sequence similarity (99.8%) to the *Escherichia coli* type strain, thereby confirming its species designation. For bladder inoculation, the strain was cultured in Luria–Bertani (LB) broth at 37 °C overnight, and the bacterial culture was adjusted to an OD_600_ (optical density of 600 nm) of 0.6–0.8 for mouse infection. Based on an established correlation for *E. coli* [33], this OD_600_ range corresponds to approximately 2–4 × 10^7^ colony-forming units (CFUs) per mL.

### 2.3. Bacterial Lysate Preparation

Cultured UPEC were centrifuged at 7200× *g* for 5 min. The pellet was resuspended in RPMI 1640 medium (GIBCO, Grand Island, NY, USA), mixed with grinding glass beads (FAKANUO, Zhuhai, China), and ground (70 Hz for 1 min, 1 min rest) for 9 cycles. After centrifugation at 2400× *g* for 5 min, the supernatant (bacterial lysate) was collected [34]. The total protein concentration of the lysate was determined using a bicinchoninic acid (BCA) assay kit (Beyotime, Shanghai, China). For stimulation experiments, MNK-3 cells were treated with the lysate at a final concentration of 50 µg/mL.

### 2.4. MNK3 Cell Culture

MNK-3 cells, an immortalized cell line with a core ILC3-like phenotype (e.g., RORγt expression, inducible IL-22 and IL-17A production) and used in prior studies of ILC3 [35,36], were utilized here as an initial, simplified model to assess changes in cytokine production following repetitive stimulation. The MNK-3 cell line used in our experiment was kindly provided by Professor Shiyang Li (Shandong University). Cells were cultured in DMEM (GIBCO, Grand Island, NY, USA), additionally supplemented with penicillin/streptomycin, and 10% FBS, with medium changed every 3 days and passaging upon reaching a density of approximately 5 × 10^5^ cells/mL.

### 2.5. Mouse Model of UTI and Experimental Design

Mice experiments were approved by the Animal Ethics Committee of the First Affiliated Hospital of Sun Yat-Sen University (animal ethics permission No. 20030J) and conducted according to the Laboratory Animal Science Association guidelines and committee requirements. 8- to 10-week-old female wildtype C57BL/6J mice were purchased from the Beijing Vital River Laboratory Animal Technology Co., Ltd. (Beijing, China). CD45.1 mice (JAX #002014) were purchased from The Jackson Laboratory (Bar Harbor, ME, USA). Experimental mice were kept in a pathogen-free environment. Each independently ventilated cage contained wood chips, and each cage contained 4–5 mice. The facilities adopt a 12-h light–dark cycle mode and operate under constant temperature and humidity conditions. Animals can eat and drink freely. The infection procedure was carried out during the eighth to tenth weeks: the water source in the cage was removed 4 h before bacterial inoculation, and the mice were then randomly divided into a control group and UTI group. Under isoflurane anesthesia, control mice received transurethral instillation of 50 µL PBS, while UTI group was infected with 50 µL of PBS containing 0.5 × 10^8^ CFU of *E. coli* CFT073 (OD_600_ 0.6–0.8) [37]. Mice were sacrificed 24 h post-infection for further analysis. Urine was collected, and bladders and kidneys were aseptically excised. The experimental unit in this study was defined as individual mice.

The establishment of UTI model was primarily confirmed by quantifying the bacterial load in bladder homogenates (CFU). The severity of infection was further evaluated through histopathological analysis of Hematoxylin and Eosin (H&E)-stained bladder sections to assess inflammation and tissue damage, complemented by monitoring body weight change and bladder weight-to-body weight ratio as indicators of systemic illness and organ-specific inflammatory response [38,39,40].

Humane endpoints were strictly defined and monitored throughout the study to minimize animal suffering. Any animal exhibiting pre-defined signs of severe distress, including severe weight loss (>20%), rapid or labored breathing, or inability to access food or water, was to be humanely euthanized immediately. No animals reached these endpoints prior to the scheduled experimental endpoint.

**Figure 1:**
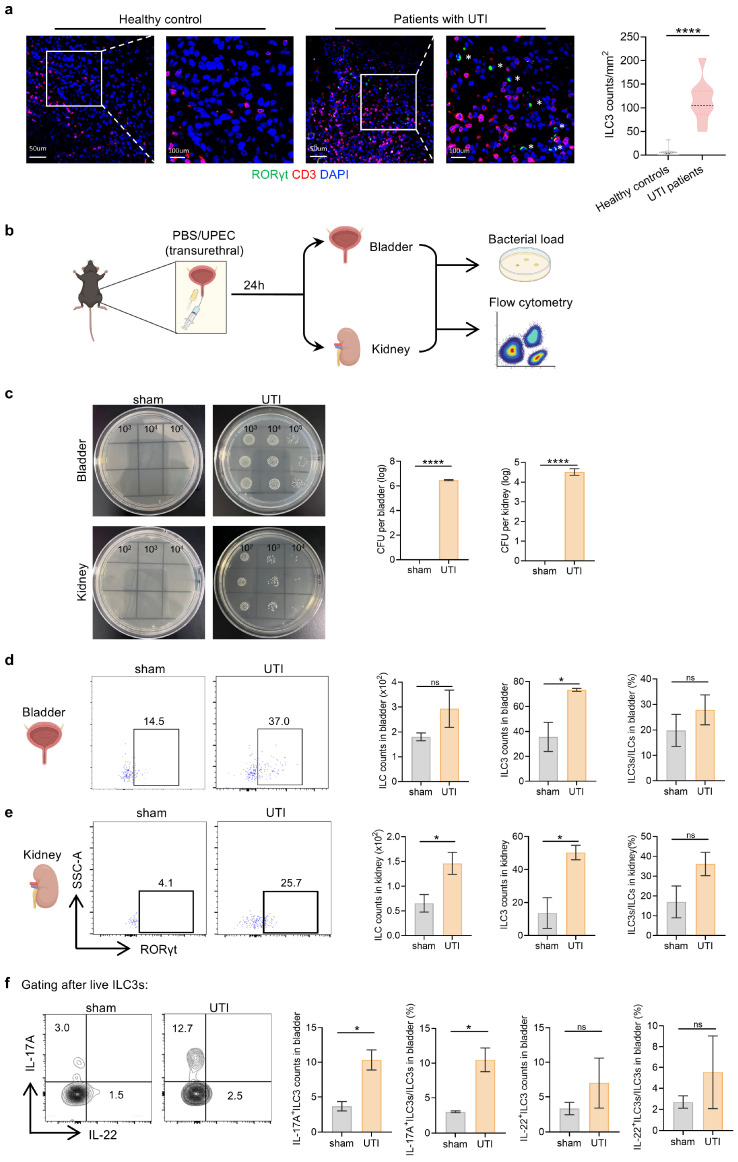
ILC3s increase systematically during UPEC-induced urinary tract infection. (**a**) Representative immunofluorescence staining and quantification of ILC3s (CD3−RORγt^+^ cells, white asterisk (*)) in human bladder sections from healthy controls (HC, *n* = 10) and UTI patients (*n* = 9). (**b**) Schematic of the mouse acute UTI model. C57BL/6 mice were transurethrally inoculated with 5 × 10^7^ CFU UPEC strain CFT073 or PBS and analyzed 24 h post-infection. (**c**) Representative images of bacterial cultures from bladder and kidney homogenates. (**d**,**e**) Representative flow cytometry plots and quantification of ILC3s in mouse bladder (**d**) and kidney (**e**). (**f**) Flow cytometry analysis of IL-17A and IL-22 expression by bladder ILC3s. Data are shown as mean ± SEM. * *p* < 0.05, **** *p* < 0.0001, ns *p* > 0.05.

**Figure 2:**
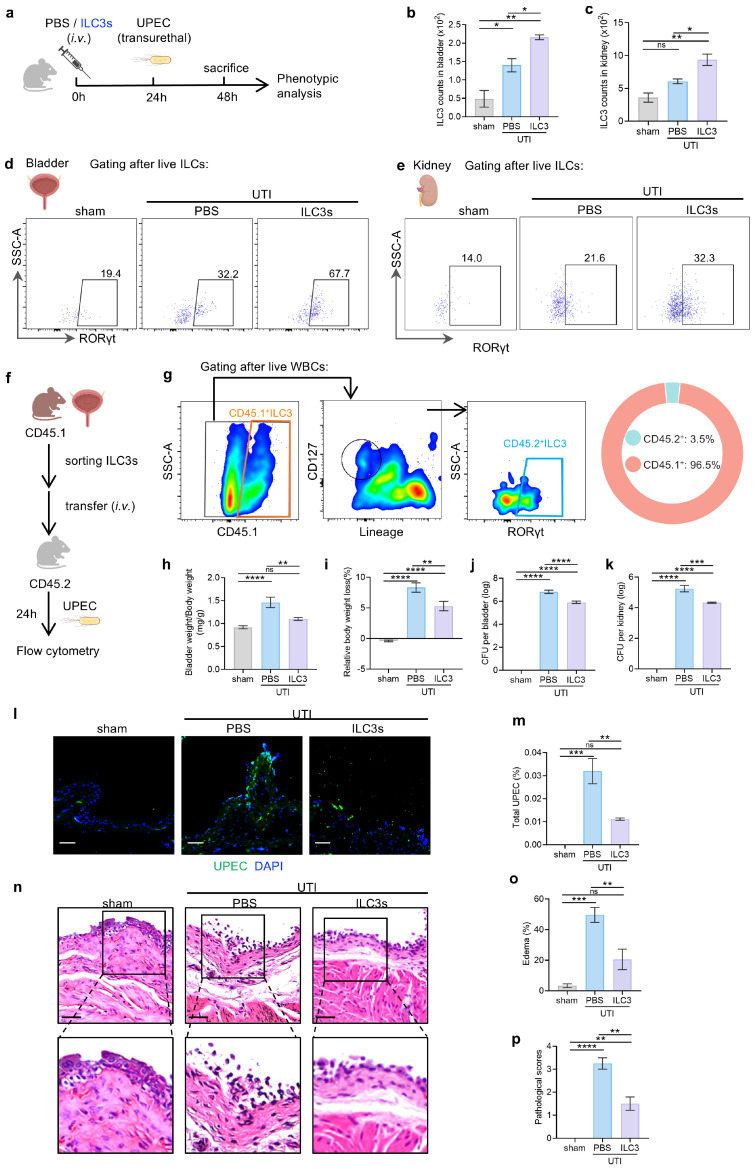
ILC3s contribute to protection against UTI. (**a**) Schematic of the adoptive transfer and infection model. (**b**–**e**) Validation of ILC3 transfer: ILC3 counts (**b**,**c**) and representative flow cytometry plots (**d**,**e**) in bladder and kidney 24 h after UPEC challenge of mice received ILC3s or PBS transfer. (**f**) Schematic of experimental design. (**g**) Flow cytometry analysis of donor (CD45.1^+^) versus host (CD45.2^+^) ILC3s in recipient bladders. The orange polygon denotes CD45.1^+^ ILC3s, the black polygon denotes CD45.2^+^ white blood cells (WBCs), the black circle denotes CD45.2^+^ ILCs, and the blue polygon denotes CD45.2^+^ ILC3s. (**h**,**i**) Disease severity assessed by bladder/body weight ratio (**h**) and body weight change (**i**). (**j**,**k**) Bacterial load of bladders (**j**) and kidneys (**k**). (**l**,**m**) Representative immunofluorescence images (**l**) and mean fluorescence intensity (MFI) quantification (**m**) of UPEC (green) in bladder mucosa. Scale bars = 50 µm. (**n**–**p**) Bladder histopathology was assessed on H&E-stained sections (**n**), scale bars = 50 µm, with quantification of submucosal edema (**o**) and pathological scores (**p**). Data are shown as mean ± SEM. * *p* < 0.05, ** *p* < 0.01, *** *p* < 0.001, **** *p* < 0.0001, ns *p* > 0.05.

**Figure 3:**
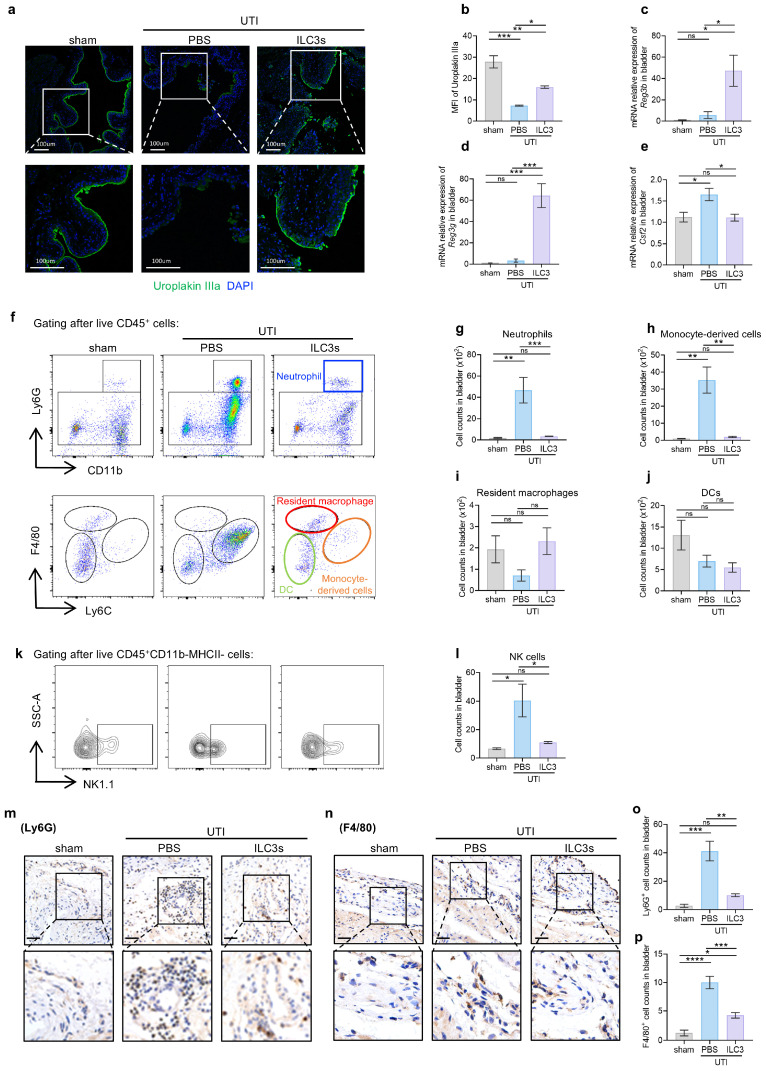
ILC3s orchestrate epithelial barrier repair and restrain inflammatory infiltration during UTI. (**a**,**b**) Representative immunofluorescence staining of Uroplakin IIIa (green) and DAPI (blue) in bladder sections and quantification of MFI. (**c**–**e**) Bladder mRNA levels of Reg3b, Reg3g, and Csf2 assessed by qPCR. (**f**–**l**) Representative flow cytometry plots and percentages of neutrophils (**g**), monocyte-derived cells (**h**), resident macrophages (**i**), dendritic cells (DC) (**j**) and natural killer cells (NK) (**l**) in bladders. In the flow cytometry plot (**f**) with CD11b on the x-axis and Ly6G on the y-axis, the upper rectangular gate represents neutrophils, and the lower rectangular gate represents Ly6G^−^ cells. In the flow cytometry plot (**f**) with Ly6C on the x-axis and F4/80 on the y-axis, the upper circle represents resident macrophages, the lower left circle represents dendritic cells (DC), and the lower right circle represents monocyte-derived cells. In the flow cytometry plot (**k**) with NK1.1 on the x-axis and SSC-A on the y-axis, the rectangular gate represents NK cells. (**m**,**n**) Representative immunohistochemical images of the Ly6G^+^ neutrophils and F4/80^+^ macrophages. Scale bar = 50 μm. (**o**,**p**) Cell counts of the Ly6G^+^ neutrophils (**o**) and F4/80^+^ macrophages (**p**) in mice bladders. Data are shown as mean ± SEM. * *p* < 0.05, ** *p* < 0.01, *** *p* < 0.001, **** *p* < 0.0001, ns *p* > 0.05.

**Figure 4:**
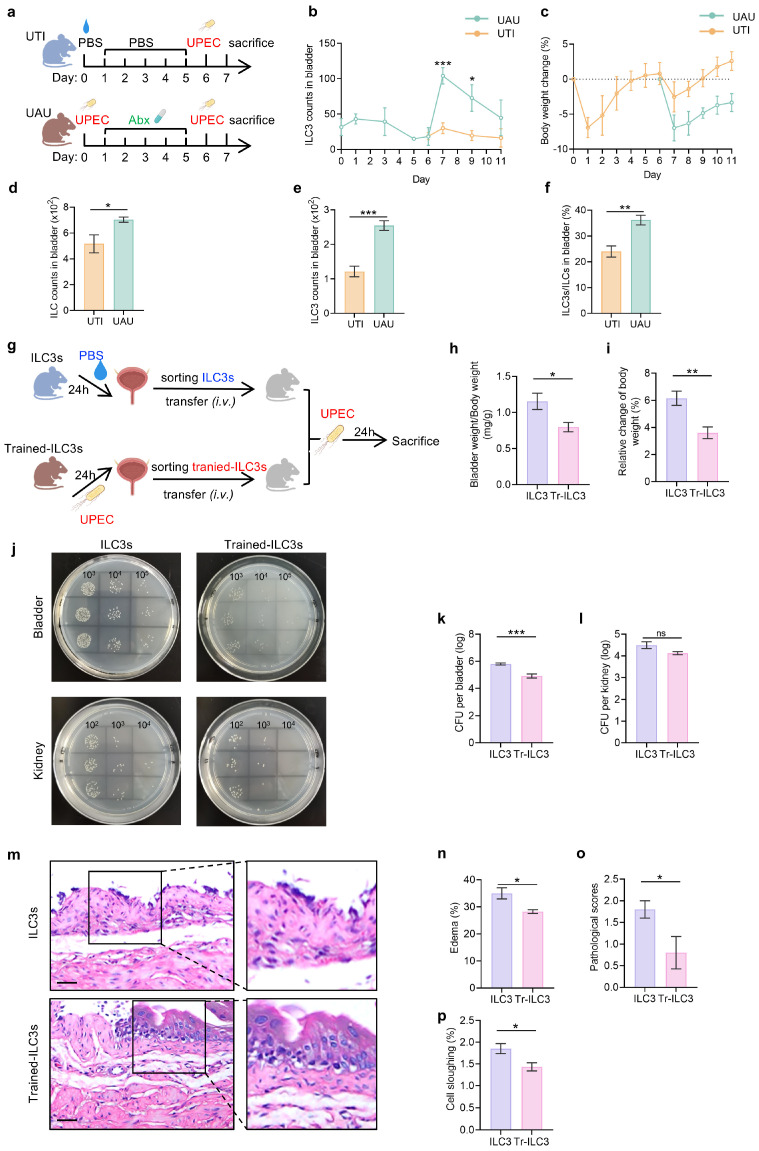
UPEC-trained ILC3s confer enhanced protection against recurrent UTI through pathogen clearance and tissue preservation. (**a**) Schematic of experimental design for primary and secondary infection. (**b**,**c**) Body weight change (**b**) and bladder ILC3 counts (**c**) monitored over the infection timeline. (**d**–**f**) ILC, ILC3 counts and the proportion of ILC3s among ILCs in bladder were detected by flow cytometry analysis. (**g**) Schematic of experimental design for adoptive transfer of naïve or Tr-ILC3s. (**h**,**i**) Disease severity in recipient mice assessed by bladder/body weight ratio (**h**) and body weight change (**i**) at 24 h post-infection. (**j**–**l**) Bacterial burden was assessed with representative culture plates (**j**) and quantified CFU in bladder (**k**) and kidney (**l**). (**m**) Representative images of H&E-stained bladder sections. Scale bars = 50 μm. (**n**–**p**) The percentage of subepidermal lamina propria edema in the urothelium (**n**), pathological scores (**o**), and the percentage of epithelial cell sloughing (**p**) were analyzed based on H&E-stained bladder sections. Data are shown as mean ± SEM. * *p* < 0.05, ** *p* < 0.01, *** *p* < 0.001, ns *p* > 0.05.

**Figure 5:**
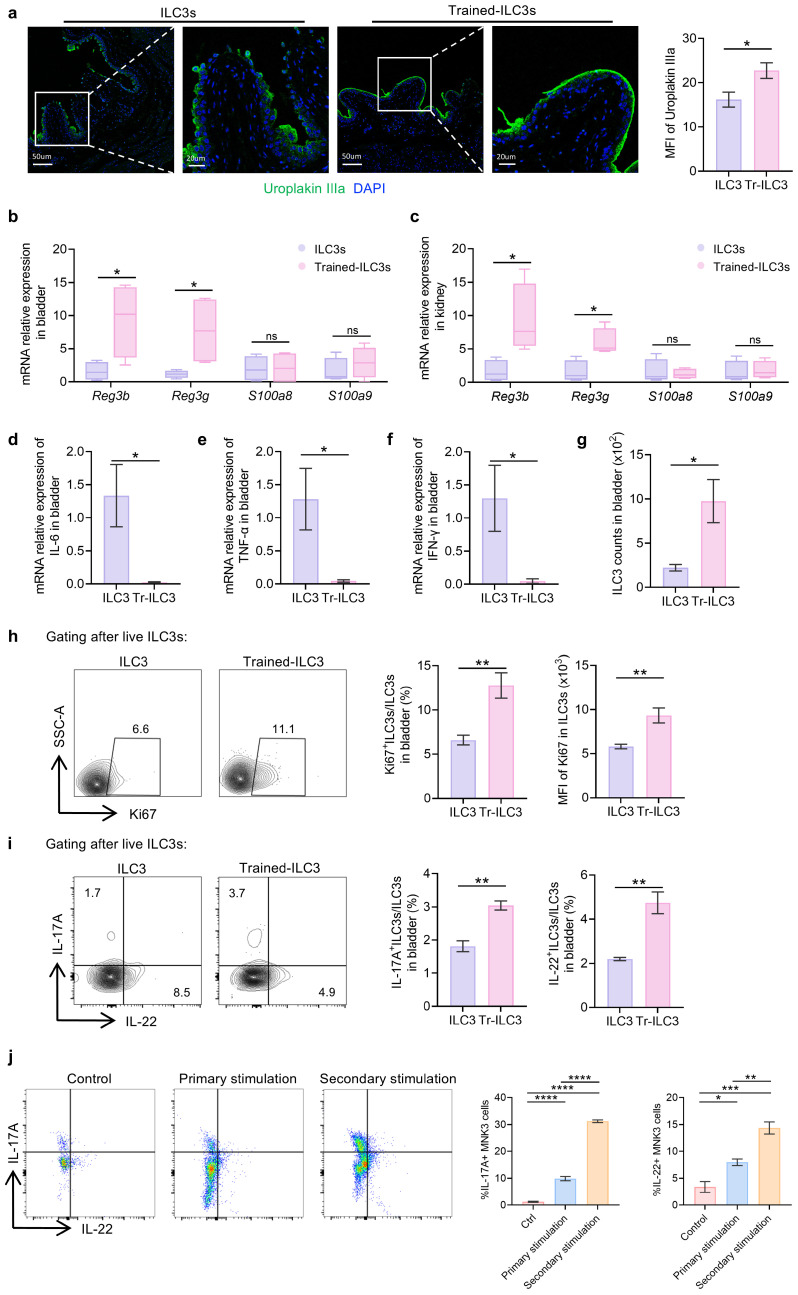
Tr-ILC3s acquire cell-intrinsic functional and proliferative advantages. (**a**) Uroplakin IIIa expression in bladder sections assessed by immunofluorescence staining (green) and MFI quantification. (**b**,**c**) The mRNA expression of *Reg3b*, *Reg3g*, *S100a8* and *S100a9* in bladders (**b**) and kidneys (**c**) was assessed by real-time qPCR. (**d**–**f**) The mRNA expression of *Il6*, *Tnfa* and *Ifng* in bladders was assessed by real-time qPCR. (**g**) ILC3 counts in bladder following transfer of naïve or Tr-ILC3s. (**h**) Proliferation of bladder ILC3s assessed by flow cytometry for Ki67 expression. (**i**) IL-17A and IL-22 expression on ILC3s in bladder were detected by flow cytometry. (**j**) IL-17A and IL-22 expression by MNK-3 cells in vitro after primary or secondary stimulation with UPEC lysate, analyzed by flow cytometry. Data are shown as mean ± SEM. * *p* < 0.05, ** *p* < 0.01, *** *p* < 0.001, **** *p* < 0.0001, ns *p* > 0.05.

Due to the limited amount of tissue obtainable from a single mouse bladder, which precludes multiple analyses on the same organ, animals within each group were randomly allocated to dedicated endpoints as follows. For the experiments presented in Figure 1, mice were randomly assigned to sham and UTI groups, with 9 mice per group (*n* = 9). Due to the small size of bladder tissue, to facilitate experimentation and ensure detection accuracy, 6 mice were randomly selected from each group for whole bladder homogenization and bacterial load (CFU) measurement, while the remaining 3 mice per group were used for whole bladder flow cytometry analysis. In the experiments shown in Figure 2 and Figure 3, mice were randomly allocated into three groups: sham, PBS + UTI, and ILC3 + UTI, with 13 mice per group (*n* = 13). In each group, 3 mice were randomly selected for whole bladder flow cytometry, 4 mice were used for quantitative PCR analysis of target genes using the whole bladder, and the remaining 6 mice were allocated as follows: half of the bladder was used for CFU assay, and the other half of the bladder was used for H&E staining and immunofluorescence staining. In experiments presented in Figure 4, for panels a–c, mice were randomly allocated to UTI and UAU (secondary UTI) groups, with 3 mice per group at different time points. For panels d–f and Figure S4, mice were randomly assigned to UTI and UAU groups, with 9 mice per group (*n* = 9). In each group, 4 mice were randomly assigned for bladder flow cytometry, and the remaining 5 mice were used for bladder H&E staining analysis. In panels g–p, mice were randomly assigned to ILC3 and trained-ILC3 (Tr-ILC3s) groups, with 5 mice per group. For Figure 5 experiments, mice were randomly divided into ILC3 and Tr-ILC3 groups, with 8 mice per group (*n* = 8). From each group, 4 mice were randomly selected for bladder flow cytometry, and the remaining 4 mice were used with half of the bladder subjected to immunofluorescence staining and the other half to mRNA analysis. We determined the sample size based on preliminary data and common practices in murine UTI studies [41,42]. A total of 155 mice were used across all experiments.

Animal inclusion and exclusion criteria were defined a priori. All mice were required to be healthy at the start of the experiment (no visible wounds or >15% non-experimental weight loss). For downstream assays, samples were excluded if they did not meet pre-established quality thresholds: RNA integrity number (RIN) < 8 for qPCR, or histology specimens with extensive tissue tearing or folding that obscured normal morphology. In accordance with the pre-defined criteria, no animals or data points were excluded from the analysis in experiments.

To minimize potential confounders, we implemented a strategy of randomization and blinding. Mice were randomly assigned to groups, and the order of all experimental procedures and sample analyses was randomized. To minimize bias, investigators were blinded to group allocation during all key outcome assessments, which were performed on coded samples.

### 2.6. Determination of Bacterial Burden

To determine total bacterial load, bladders were homogenized in 1 mL PBS. After serial dilutions, the suspension was spread on LB agar plates. After overnight incubation in a 37 °C thermostat, the colony forming units on the plates were counted and analyzed.

### 2.7. Isolation of Leukocytes from Bladders and Kidneys

The preparation of cell suspensions follows established methods [43]. Bladders or kidney tissues were cut into small pieces about 1 mm, washed with sterile PBS. Bladders were digested in RPMI 1640 medium (GIBCO, Grand Island, NY, USA) supplemented with DNase I (100 µg/mL, Roche, Penzberg, Germany), Liberase TM (0.34 Units/mL, Roche, Mannheim, Germany), and 2% FBS at 37 °C for 60 min, with intermittent manual agitation at 15-min intervals. Kidneys were digested in RPMI 1640 medium (GIBCO, Grand Island, NY, USA) supplemented with 0.05% Dispase (GIBCO, Grand Island, NY, USA), 0.1% Type II Collagenase (GIBCO, Grand Island, NY, USA), and 2% FBS at 37 °C for 40 min. Then the digested tissues were filtered through a cell strainer (70 μm) to obtain single-cell suspensions.

### 2.8. Flow Cytometry Analysis

Here is a compilation of antibodies we used for flow cytometry. Data were collected on BD Fortessa (BD Biosciences, San Jose, NJ, USA) and Attune NxT (ThermoFisher, Eugene, OR, USA) instruments, and then analyzed through FlowJo version 10. To exclude dead cells, the fixable viability dyes eFluor780 or eFluor506 (Thermo Fisher, Carlsbad, CA, USA), Brilliant Violet 510™ (BD Biosciences, San Jose, NJ, USA) were used.

The cells were stained in the dark at 4 °C using the following antibody cocktails: from Biolegend (San Diego, CA, USA), CD45 (clone 30-F11, APC, Cat#103112), CD45 (clone 30-F11, APC/Fire™ 750, Cat#103154), Lineage Cocktail with Isotype Ctrl (including anti-mouse CD3ε, clone 145-2C11; anti-mouse Ly-6G/Ly-6C, clone RB6-8C5; anti-mouse CD11b, clone M1/70; anti-mouse CD45R/B220, clone RA3-6B2; anti-mouse TER-119/Erythroid cells, clone Ter-119, FITC, Cat#133302), CD127 (clone A7R34, PE/Cy7, Cat#135014), CD127 (clone A7R34, Brilliant Violet 421™, Cat#135023), CD117 (clone 2B8, PE, Cat#105808), CD117(clone 2B8, APC/Cy7, Cat#105826), CD45.1 (clone A20, APC, Cat#110714), IL-17A(clone TC11-18H10.1, Brilliant Violet 421™, Cat#506906), F4/80 (clone BM8, PE/Dazzle™ 594, Cat#123146), Ki67 (clone 12B2B64, BV605, Cat#652413),from Thermo Fisher Scientific, ROR Gamma T (clone B2D, APC, Cat#17698182), IL-22(clone 1H8PWSR, PE, Cat#12722182), NK1.1 (clone PK136, SB780, Cat#78594182), from BD Biosciences, IL-17A (clone TC11-18H10, AF647, Cat#560184).

### 2.9. Detection of Cytokines in ILCs

To detect intracellular cytokines and transcription factors, single cell suspensions were stimulated for 4 h in vitro with Cell Stimulation Cocktail (plus protein transport inhibitors) (eBioscience, San Diego, CA, USA, Cat# 00497593), diluted to 1× and incubated at 37 °C. Cells underwent surface marker staining, followed by permeabilization and subsequent intracellular staining for cytokines and transcription factors with the Transcription Factor Staining Buffer Set (eBioscience, Cat#00512343, Cat#00522356, Cat#00552300).

### 2.10. Adoptive Transfer of ILC3s

Bladder cells were isolated from sham and UPEC-infected mice. Single-cell suspensions were immunostained and applied to high-purity cell sorting for CD45^+^Lineage^−^CD127^+^CD117^+^ cells on a FACSAria II Cell Sorter (BD Life Sciences, Milpitas, CA, USA). Then, 3–6 × 10^3^ cells were transferred intravenously into recipient mice.

### 2.11. In Vivo Neutralization of IL-17A and IL-22

To functionally assess the ILC3-mediated protection mediated by IL-17A and IL-22, neutralizing antibodies against these cytokines were administered in vivo. Specifically, mice that had received adoptive transfer of ILC3s were intraperitoneally (i.p.) injected with anti-IL-17A neutralizing antibody (MAB421-100, R&D, Minneapolis, MN, USA) or anti-mouse IL-22 neutralizing antibody (AF582, R&D). Each antibody was administered at a dose of 80 μg per mouse. Injections were performed after UPEC challenge. Control groups received equivalent injections of isotype antibody (MAB002, R&D) at the same dose [44].

### 2.12. Hematoxylin and Eosin (H&E) Staining

Bladder tissue was fixed in 4% PFA, paraffin-embedded, and cut into 5-μm sections. Slides were dewaxed, hydrated and subjected to hematoxylin and eosin staining following standard protocols. Bladder histopathological scores (over each section’s total area) were determined by previously described criteria [45,46,47,48].

### 2.13. Immunofluorescence Staining (IF) and Immunohistochemistry (IHC)

For IF, both paraffin and frozen sections were permeabilized and blocked (0.2% Triton X-100, 10% donkey serum) prior to incubation with primary antibodies against Uroplakin III (Abcam, Cambridge, UK, ab231576), RORγt (eBioscience, San Diego, CA, USA, 14-6988-82), and CD3 (Abcam, Cambridge, UK, ab5690) overnight at 4 °C. The sections were washed and subjected to secondary antibody incubation (Alexa Fluor 546 anti-rat, 647 anti-rabbit, 488 anti-mouse; Thermo Fisher, Eugene, OR, USA) for 1 h. Nuclei were stained with DAPI (Thermo Fisher, Eugene, OR, USA) for 5 min. Images were acquired via confocal laser scanning microscopy (Zeiss 880, Jena, Germany). Mean fluorescence intensity was quantified with Zeiss Zen software (ZEN 2.3, blue edition).

For IHC, bladder paraffin sections were deparaffinized and subjected to high-pressure sodium citrate antigen retrieval. Endogenous peroxidase was inhibited with 3% H_2_O_2_ for 10 min, followed by blocking with 3% BSA at room temperature (RT) for 15 min. Sections were incubated with primary antibody (MCA497G) for 1 h at RT, then secondary antibody for 30 min at RT, and finally stained with DAB (3,3′-Diaminobenzidine) and hematoxylin.

### 2.14. RNA Extraction and Reverse Transcription

At 24 h post-infection, bladder tissues were homogenized manually and processed for RNA isolation. Total RNA was extracted using the RNeasy Mini kit (Qiagen, Hilden, Germany, 74106) following the supplier’s guidelines. Subsequently, cDNA was synthesized from up to 0.5 μg RNA using the High-Capacity cDNA Reverse Transcription Kit (Applied Biosystems, Foster City, CA, USA).

### 2.15. Reverse Transcription-Quantitative Polymerase Chain Reaction (RT-qPCR)

Primers were designed using Primerbank 3.0 (https://pga.mgh.harvard.edu/primerbank/, accessed on 21 April 2021) and verified with Primer-BLAST (August 2020 updated version) (https://www.ncbi.nlm.nih.gov/tools/primer-blast/, accessed on 21 April 2021). Real-time PCR reactions were prepared with qPCR SYBR Green Master Mix (LightCycler 480 SYBR Green I Master, 4887352001-1) (Roche, Indianapolis, IN, USA) per instructions and run on a QuantStudio™ 7 Flex system (Applied Biosystems). The 2^−ΔΔCT^ method was employed to calculate relative gene expression. All the primers used in this study are mentioned in Table S1.

### 2.16. Statistical Analysis

All data are shown as mean ± SEM and were analyzed with GraphPad Prism 9. Group differences were assessed by unpaired two-tailed *t*-test (two groups) or one/two-way ANOVA with Bonferroni correction (multiple groups). Significance is labeled as * *p* < 0.05, ** *p* < 0.01, *** *p* < 0.001 (ns, *p* > 0.05).

## 3. Results

### 3.1. ILC3s Increase Systematically During UPEC-Induced Urinary Tract Infection

To determine the alteration of ILC3s in UTIs, immunofluorescence staining of bladder sections from 9 UTI patients and 10 healthy controls (HCs) (Table S2) revealed a remarked accumulation of CD3^−^RORγt^+^ ILC3s in UTI patients compared to HCs (Figure 1a). To further explore the dynamics and role of ILC3s in UTI, we established a murine UTI model by transurethral inoculation with UPEC strain CFT073, a validated approach for inducing ascending cystitis and pyelonephritis [37]. Sham-operated controls received equivalent PBS volumes. Bladders and kidneys were analyzed 24 h post-infection (Figure 1b). While no bacteria were detected in either the bladder or the kidney in sham controls, the UPEC-infected mice exhibited a notable rise in CFUs, surpassing 10^6^ in the bladder and exceeding 10^4^ in the kidney, signifying the successful induction of the UTI model (Figure 1c).

We next examined the changes in ILCs and ILC3s upon infection. ILCs were defined as live CD45^+^Lin^−^CD127^+^ lymphocytes, and ILC3s were defined as RORγt^+^ ILCs (Figure S1). Flow cytometric analysis revealed a remarkable increasing tendency of absolute counts of ILC3s in the bladders, and both the number of ILC3 cells and the proportion of ILC3 among ILCs in the kidneys were significantly elevated, while total ILCs levels remained stable across groups (Figure 1d,e). Given that IL-17A and IL-22 are major effectors in ILC3-mediated defense against infections, we assessed the functional characterization [30,49,50]. A more than two-fold increase in IL-17A^+^ ILC3s was detected in the bladder of UTI mice, whereas IL-22 production showed no difference (Figure 1f).

Altogether, these findings establish that UPEC infection triggers expansion of IL-17A-skewed ILC3s within the urinary tract mucosa, suggesting their specialized role in early antimicrobial defense.

### 3.2. ILC3s Contribute to Protection Against UTI

Recent studies have reported that ILC3-deficient mice exhibit increased bacterial burden after UPEC infection, suggesting that ILC3s are required for resistance to UTIs [22]. To directly assess the protective role of ILC3s in UTIs, we employed an adoptive transfer strategy in which purified bladder-derived ILC3s were intravenously administered to recipient mice (Figure 2a). Flow cytometric tracking confirmed successful engraftment, with donor-derived ILC3s constituting >90% of total bladder ILC3s at 24 h post-transfer (Figure 2b–g). We also confirmed the purity of the sorted cells prior to transfer by intracellular staining for the lineage-defining transcription factor RORγt. Within re-gated ILCs, 84.0% of cells expressed RORγt, identifying them as canonical ILC3s (Figure S2b), demonstrating that the transferred cells were a highly enriched population of ILC3s. Recipient mice challenged with UPEC exhibited less body exhaustion and reduced bladder edema compared to PBS-treated group (Figure 2h,i). Quantification of bacterial burdens revealed that ILC3 transfer reduced bladder and kidney CFUs by 10-fold (Figure 2j,k and Figure S2). Immunofluorescence staining confirmed a notable reduction in urothelial UPEC colonization in ILC3 recipient mice (Figure 2l,m), consistent with enhanced luminal pathogen clearance.

Histopathological analysis further validated these findings. ILC3 transfer significantly reduced the severe bladder damage exhibited in untreated UTI mice, including epithelial denudation, marked lamina propria edema, and dense perivascular leukocyte infiltration, while restoring near-normal mucosal architecture (Figure 2n–p). These data demonstrate that exogenous ILC3s provide multi-layered protection against UPEC infection by enhancing bacterial clearance in the bladder and kidney regions and maintaining urothelial barrier integrity, underscoring their pivotal role in UTI defense.

### 3.3. ILC3s Orchestrate Epithelial Barrier Repair and Restrain Inflammatory Infiltration During UTI

UPEC adheres to and invades urothelial cells, causing epithelial cell death and exfoliation, followed by further bacterial replication and inflammatory cell recruitment [51]. To dissect the ILC3s’ role in mucosal repair and immune regulation, we first analyzed urothelial barrier restoration. Immunofluorescence quantification revealed a 73% reduction in Uroplakin IIIa (Upk3a)—a critical structural protein for umbrella cells —in UPEC-infected bladders [52,53]. ILC3s transfer restored Upk3a expression and epithelium integrity to a level surpassing half of the baseline (Figure 3a,b), indicating robust epithelial repair. Considering that intestinal ILC3s facilitate bacterial clearance and epithelial repair by antibacterial peptides (AMPs) production [50], subsequent analyses delved into the expression of two main AMPs in bladders, Reg3b and Reg3g. The transcripts of these AMPs in bladders were barely detectable at steady or post-infected state, while they were induced by ILC3s transfer (Figure 3c,d).

We next interrogated ILC3-mediated immunomodulation. UPEC infection induced a marked increase in pro-inflammatory cytokines, including *Il1b*, *Tnfa*, and *Il6*, which declined nearly to the initial level after ILC3s transfer (Figure S3a–c). Concomitant reduction in *Csf2* transcripts in the bladder of ILC3-transferred mice suggested attenuated granulocyte-macrophage colony-stimulating factor (GM-CSF)-dependent myeloid recruitment (Figure 3e). Immune profiling (Figure S3d,e) confirmed this anti-inflammatory phenotype, with elevated neutrophil, monocyte-derived cell, and NK cell infiltration in UTI mice compared to sham controls, which was reduced by ILC3 transfer to a level approaching pre-infection state, whereas resident macrophages and dendritic cells remained unaffected (Figure 3f–l). Accordingly, immunohistochemical staining showed ILC3 transfer significantly reduced neutrophil infiltration in the lamina propria and macrophage density in perivascular regions (Figure 3m–p).

### 3.4. UPEC-Trained ILC3s Confer Enhanced Protection Against Recurrent UTI Through Pathogen Clearance and Tissue Preservation

In order to explore the role of ILC3-trained immunity in recurrent UTI, we developed a sequential infection model wherein mice were treated with ciprofloxacin (80 mg/kg, once daily by oral gavage) from day 1 to day 5 post-infection to achieve complete bacterial clearance [30,54,55]. Quantitative bacterial culture of bladder homogenates from antibiotic-treated mice, performed to confirm tissue-level clearance for the subsequent challenge, confirmed undetectable bacterial loads. These cured mice (“UAU”) or naïve controls (“UTI”) were then re-challenged with UPEC to simulate clinical recurrence (Figure 4a). ILC3 dynamic analysis revealed that primary infection triggered a transient expansion of bladder ILC3s, which returned to baseline levels post-antibiotic treatment. Remarkably, secondary infection caused a surge in ILC3 abundance compared with primary infection, followed by a gradual contraction to stable levels (Figure 4b). This amplified response paralleled improved clinical outcomes, with UAU mice exhibiting significantly reduced weight loss and bladder edema compared to UTI controls (Figure 4c and Figure S4a,b).

At 24 h post-rechallenge, secondary infection mice demonstrated significant immunological remodeling: total bladder ILCs increased over 30%, with ILC3s constituting 36% of the population versus 24% in primary infection (Figure 4d–f). Histopathological assessment confirmed enhanced protection, showing a reduction in epithelial denudation and less lamina propria edema in UAU mice (Figure S4c–e). To further define the functional state of these cells, we tracked cytokine production in bladder ILC3s throughout the infection and resolution timeline. During the acute phase of primary infection, ILC3s showed a marked induction of IL-17A and IL-22. Then cytokine production returned to baseline levels following antibiotic clearance and prior to secondary challenge (Figure S4g,h). This functional quiescence after resolution followed by a robust recall response aligns with a trained immunity phenotype rather than sustained inflammation [56,57].

We thus hypothesized that ILC3s exposed to primary infection, here named as trained-ILC3s (Tr-ILC3s), could acquire enhanced defensive capacity against UPEC rechallenge. To ascertain the contribution of Tr-ILC3s in the reinfection, we performed adoptive transfer experiments. Bladder-derived ILC3s from infection-resolved mice (Tr-ILC3s) or naïve donors were transferred to recipient mice prior to UPEC challenge (Figure 4g). Tr-ILC3s recipients exhibited superior protection, demonstrating attenuated weight loss, reduced bladder-to-body weight ratio, and lower bacterial burden in the bladder compared to naïve ILC3s recipients (Figure 4h,l). Histological analysis revealed that Tr-ILC3s mitigated hallmark UTI pathology, reducing surface cell shedding, lamina propria edema, and perivascular leukocyte infiltration compared to naïve ILC3 recipients (Figure 4m–p). To further substantiate that the protective effect is intrinsic to ILC3s and independent of adaptive immunity, we performed parallel adoptive transfer experiments in Rag1-deficient mice, which lack mature T and B lymphocytes [58]. Tr-ILC3s from Rag1^−^/^−^ mice transferred into Rag1^−^/^−^ recipients still conferred superior protection compared to naïve ILC3s, as evidenced by a significantly lower bladder bacterial burden (CFU) (Figure S4i,j).

### 3.5. Tr-ILC3s Acquire Cell-Intrinsic Functional and Proliferative Advantages

To elucidate the mechanistic basis of ILC3-trained immunity, we systematically compared the functional and proliferative capacity of Tr-ILC3s versus naïve counterparts during UPEC rechallenge. Tr-ILC3 recipients exhibited superior epithelial repair, with mean fluorescence intensity (MFI) of Upk3a expression of Tr-ILC3 recipients significantly higher than naïve ILC3 recipients (Figure 5a). Antimicrobial defense was also markedly enhanced, as Tr-ILC3s induced higher expression of *Reg3b* and *Reg3g* over six-fold in the bladder and kidneys compared to naïve ILC3s. Notably, *S100a8/a9* expression—a marker of neutrophil-driven inflammation—remained unchanged, confirming the specificity of ILC3-mediated AMP regulation (Figure 5b,c). Tr-ILC3s profoundly attenuated inflammatory cascades, reducing *Il6*, *Tnfa*, and *Ifng* to a hardly detectable level compared to naïve ILC3 controls (Figure 5d–f).

This anti-inflammatory phenotype correlated with a substantial increase in the number of ILC3s, as well as both the proportion and MFI of Ki67^+^ cells within the Tr-ILC3s, relative to the naïve ILC3s (Figure 5g,h, gating strategy shown in Figure S5a). Functional superiority was further evidenced by greater IL-17A and IL-22 production in Tr-ILC3s versus naïve cells (Figure 5i), which is indicative of a memory response to a secondary challenge. To functionally confirm the protection mediated by IL-17A and IL-22, we performed in vivo neutralization experiments. The adoptive transfer of ILC3s significantly reduced bladder bacterial burden compared to controls, recapitulating their protective effect. Crucially, this protection was markedly attenuated when mice receiving ILC3s were co-administered neutralizing antibodies against either IL-17A or IL-22, as evidenced by a significant rebound in tissue CFU (Figure S5b). These data provide direct functional evidence that the enhanced IL-17A and IL-22 production by Tr-ILC3s is mechanistically required for their superior antimicrobial defense.

We further utilized an in vitro ILC3 model, the MNK-3 cell line, to investigate whether trained immunity induced by UPEC lysate stimulation could lead to functional changes, with the gating strategy shown in Figure S5c [35]. The percentage of IL-17A^+^ MNK-3 and IL-22^+^ MNK-3 upon secondary stimulation was significantly higher than that upon primary stimulation (Figure 5j), which was consistent with the in vivo results, underscoring that UPEC can directly modulate and reshape ILC3 responsiveness to the secondary challenge.

## 4. Discussion

This study unveils a novel mechanism in urinary tract immunity: the induction of trained immunity in bladder-resident ILC3s following UPEC exposure. We provide the first comprehensive evidence that these “Tr-ILC3s” acquire enhanced, cell-intrinsic defensive capabilities, leading to superior protection against rUTIs. Our findings demonstrate that UPEC rechallenge specifically triggers an amplified and functionally polarized expansion of IL-17A- and IL-22-producing ILC3s, which critically contribute to durable protection by reinforcing urothelial barrier integrity through Reg3b/Reg3g upregulation and effectively restraining detrimental inflammation (e.g., IL-6 production). The therapeutic potential of this mechanism is underscored by our adoptive transfer experiments, where Tr-ILC3s significantly alleviated recurrent infection, establishing immunity as a previously unrecognized and highly promising pathway for sustained mucosal defense. This study directly addresses the core clinical-immunological contradiction of rUTIs—the failure of initial infection to confer reliable protection against recurrence—by providing a mechanistic explanation for how the innate immune system develops memory to prevent repeated infections. This represents a significant conceptual advance in the field of urinary tract immunology.

While previous work, notably by Huang et al. (2022), established the necessity of ILC3s for host resistance to acute UPEC infection by demonstrating increased bacterial burden in ILC3-deficient mice, our study extends this understanding significantly by focusing on the adaptive, memory-like properties of ILC3s in the context of recurrent UTIs [22]. Huang et al. primarily investigated the role of ILC3s in the initial immune response and their general contribution to bacterial clearance. In contrast, our work specifically identifies and characterizes the phenomenon of trained immunity in bladder ILC3s, demonstrating that prior UPEC exposure fundamentally reprograms these cells to mount a superior and more efficient protective response upon subsequent challenge. Our time-course analysis further refines the understanding of ILC3 dynamics, showing a rapid yet transient expansion post-primary infection, followed by a marked and amplified surge upon secondary challenge—a hallmark of trained immunity that was not explored in previous acute infection models. This amplified response, coupled with enhanced IL-17A and IL-22 production, distinguishes the ‘trained’ ILC3s and highlights their unique contribution to long-term defense against recurrence.

The comprehensive protective effects of Tr-ILC3s—encompassing bacterial clearance, barrier integrity, and inflammation control—suggest a highly coordinated and effective innate memory response capable of addressing multiple facets of UTI pathogenesis and recurrence [24,59,60,61]. This multi-pronged attack makes the Tr-ILC3 response particularly robust and therapeutically attractive. Our study clarifies the multiple mechanisms by which Tr-ILC3s contribute to host protection. To start with, these cells enhance pathogen clearance, evidenced by significantly reduced bacterial burdens in recipient mice. Complementing this antimicrobial activity, Tr-ILC3s reinforce urothelial barrier integrity through Upk3a restoration and potent induction of antimicrobial peptides—notably elevating Reg3b/Reg3g expression over six-fold higher than in naïve ILC3s. This establishes an “ILC3-cytokine-AMP axis” in the bladder, where Tr-ILC3s upregulate IL-17A and IL-22 secretion, thereby driving epithelial cells to produce antimicrobial peptides. This epithelial-centric mechanism contrasts with ILC3 functions in other mucosal sites, highlighting tissue-specific adaptations critical for UTI defense [15,16,19,26,30]. Concurrently, Tr-ILC3s actively restrain inflammation by suppressing the expression of pro-inflammatory cytokines (e.g., *Il1b*, *Tnfa*, and *Il6*) and diminishing the recruitment of pathogenic myeloid cells. This balanced protective response helps avoid immunopathology.

An intriguing observation from our experimental series was the apparent dissociation between neutrophil infiltration and S100a8/a9 mRNA levels. It is important to note that these readouts originated from two distinct but complementary comparisons. First, the significant reduction in bladder neutrophils in naïve ILC3-recipient mice compared to PBS recipients demonstrates the potent intrinsic capacity of adoptively transferred ILC3s to mitigate acute, neutrophil-driven inflammation. Second, the comparable levels of S100a8/a9 mRNA between mice receiving naïve ILC3s versus Tr-ILC3s suggest that the enhanced protection conferred by training may not simply amplify this anti-inflammatory effect. This can be reconciled by considering the cellular origins of S100a8/a9 in the bladder. Beyond neutrophils, the urothelium itself is also a documented source of these proteins during urinary tract infection [62]. Therefore, the total tissue S100a8/a9 signal likely represents a composite of contributions from both infiltrating immune cells and resident epithelial cells. We propose that the unchanged overall mRNA level in the context of reduced neutrophilic inflammation may reflect a shift in its cellular source—a decrease from neutrophils potentially accompanied by a modulated output from the urothelium as Tr-ILC3s promote mucosal repair and barrier homeostasis. This interpretation aligns with our functional data showing superior epithelial repair in the Tr-ILC3 group and collectively indicates that the trained phenotype may pivot towards enhancing tissue resilience after the initial inflammatory insult is effectively controlled.

Our demonstration of the superior efficacy of Tr-ILC3s provides a novel conceptual foundation for future UTI therapeutic strategies. This finding is notable given the escalating crisis of multidrug-resistant uropathogens, where conventional antibiotic strategies are increasingly failing to prevent recurrence [63]. By harnessing the intrinsic memory of the innate immune system, ILC3-based therapies may offer a complementary strategy for infection management, moving beyond direct pathogen targeting to bolster host resilience. Translating these findings, future studies exploring therapeutic translation might consider several complementary approaches. First, ex vivo expansion and adoptive transfer of autologous Tr-ILC3s may benefit patients with severe or refractory rUTIs. Second, pharmacological or immunological conditioning aimed at promoting a trained phenotype in bladder-resident ILC3s represents another investigative avenue. Third, integrating ILC3-modulating approaches with existing or emerging treatments like phage therapy or vaccines might be explored for synergistic protection. This work contributes to the emerging field of host-directed immunotherapy for infectious diseases. If antibiotic therapy is failing, such a host-centric approach may represent a distinct shift in perspective in recurrent infection management.

While the therapeutic potential of harnessing ILC3-trained immunity is compelling, its clinical translation faces several formidable challenges that must be acknowledged. First, translating in vitro ILC3 generation into a robust manufacturing process is a major hurdle. While protocols exist to derive ILC3s from hematopoietic progenitors in specialized culture systems, scaling these processes to produce therapeutic cell doses that reliably recapitulate the functional and phenotypic properties of tissue-resident ILC3s presents significant challenges in standardization, quality control, and cost-effectiveness [64,65]. Second, ensuring effective tissue targeting and residency is critical. Systemically infused ILC3s may not efficiently home to and persist in the bladder mucosa; thus, routes of administration and strategies to enhance homing require careful evaluation. Third, and paramount, are safety considerations. The potential for excessive or off-target inflammation must be addressed, given the complex roles of ILC3-derived cytokines. Notably, human ILC3s exhibit varying sensitivity to common immunosuppressants, which complicates risk control [66]. Finally, patient heterogeneity presents a major translational variable. The baseline immune status and bladder microenvironment likely vary greatly among individuals with rUTI, which could influence efficacy and necessitate the identification of predictive biomarkers. Addressing these challenges will be indispensable for moving this promising concept from bench to bedside.

Despite the foundational insights into ILC3-trained immunity in UTIs, several limitations should be acknowledged. First, all human tissues were obtained from an oncological setting; while this allowed for a controlled comparison, future validation in tissues from patients with non-neoplastic urological conditions is crucial to confirm the generalizability of these mechanisms. Second, we explicitly acknowledge that our definition of the “trained” phenotype is based on durable functional enhancement (recall proliferation and cytokine responses) after the resolution of primary infection. While the canonical definition of trained immunity includes epigenetic and metabolic reprogramming [59], direct profiling of these molecular layers in rare tissue-resident ILC3s remains a technical challenge due to the extremely limited cell numbers obtainable from mouse bladder tissue. Third, regarding the MNK-3 cell line, while useful for studying ILC3 in vitro [35,36], t cannot fully recapitulate the complexity of tissue-resident primary ILC3s, as it lacks the tissue-specific microenvironment and potential heterogeneity of bladder ILC3s. Therefore, observations made in MNK-3 cells served solely to generate a testable hypothesis regarding cytokine induction. The central findings of this work—that ILC3s can acquire a trained, protective memory phenotype—are conclusively supported by our in vivo data. Fourth, this study used female mice to model UTI. This choice is justified by the higher clinical incidence of UTI in women and the common and classic use of female mice in experimental UTI research [67,68,69]. However, we acknowledge that this limits the generalizability of our findings to males. In summary, our work establishes a functional and phenotypic framework for future mechanistic dissection.

Building on this foundation, future priorities should address several interconnected fronts. Defining the molecular basis of UPEC-induced ILC3 reprogramming—including key pathogen-derived signals and their epigenetic-metabolic consequences—would provide crucial insights for therapeutic harnessing of this trained immunity. Simultaneously, translational refinement of ILC3-based strategies requires optimization of ex vivo expansion protocols, targeted delivery systems, and thorough assessment of safety and effectiveness. Concurrently, synergistic potential with emerging anti-UTI modalities (e.g., novel antimicrobials, phage therapy, or vaccines) warrants systematic exploration to combat antibiotic resistance. Ultimately, clinical translation necessitates validation in human cohorts and identification of ILC3 training biomarkers for patient stratification—collectively advancing toward precision interventions for recurrent UTIs.

## 5. Conclusions

Our findings establish ILC3-trained immunity as a critical defense mechanism against UTIs and highlight the therapeutic potential of ILC3-based strategies. By elucidating the role of Tr-ILC3s in mucosal immunity, this study paves the way for innovative approaches to combat recurrent and antibiotic-resistant UTIs, representing a significant advancement in our understanding of uropathogen management.

## Data Availability

The original contributions presented in this study are included in the article/Supplementary Materials. Further inquiries can be directed to the corresponding author(s).

## Supplementary Materials

The following supporting information can be downloaded at: https://www.mdpi.com/article/10.3390/biomedicines14010078/s1, Figure S1: Gating strategy used to identify ILC3s in mice; Figure S2: Representative images of bacterial load of bladders and kidneys in Figure 2j,k; Figure S3: Bladder inflammatory cytokine expression and immune cell gating strategies; Figure S4: Evaluation of disease severity and bladder histopathology post-secondary infection; Figure S5: Evaluation of disease severity and bladder histopathology post-secondary infection; Table S1: The primer sequences of target genes; Table S2: Clinical characteristics of healthy controls and UTI patients detected for renal ILC3s by immunofluorescence.

## Abbreviations

The following abbreviations are used in this manuscript:

UTI	Urinary tract infection
ILC	Innate lymphoid cell
ILC3	Group 3 innate lymphoid cell
UPEC	Uropathogenic *Escherichia coli*
ESRD	End-stage renal disease
ICU	Intensive care unit
BCG	Bacille Calmette-Guérin
IL-17A	Interleukin-17A
IL-22	Interleukin-22
Upk3a	Uroplakin IIIa
CFU	Colony-forming units
AMP	Antibacterial peptides
HC	Healthy controls
