# Supplementary material for: Trained Immunity in Bladder ILC3s Enhances Mucosal Defense Against Recurrent Urinary Tract Infections
Source: Biomedicines. 2025 Dec 30;14(1):78. doi: 10.3390/biomedicines14010078 (PMC12837945; doi:10.3390/biomedicines14010078)
Supplement: Supplementary file 1 [file biomedicines-14-00078-s001.zip › biomedicines-3965099-supplementary.pdf]

Supplementary Materials

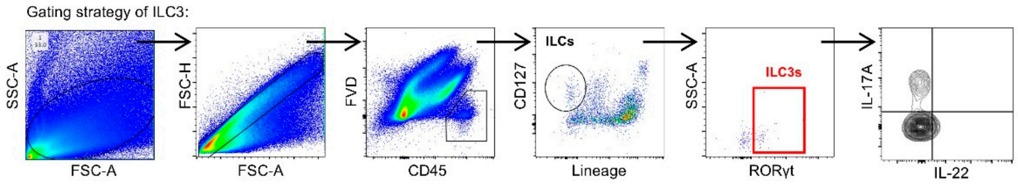

Figure S1. Gating strategy used to identify ILC3s and IL-17A/IL-22 expression. ILCs were defined as CD45+Lineage-CD127+ cells, and ILC3s were defined as RORγt+ ILCs. (Lineage=CD3ε, B220, CD11b, Gr-1, and Ter-119).

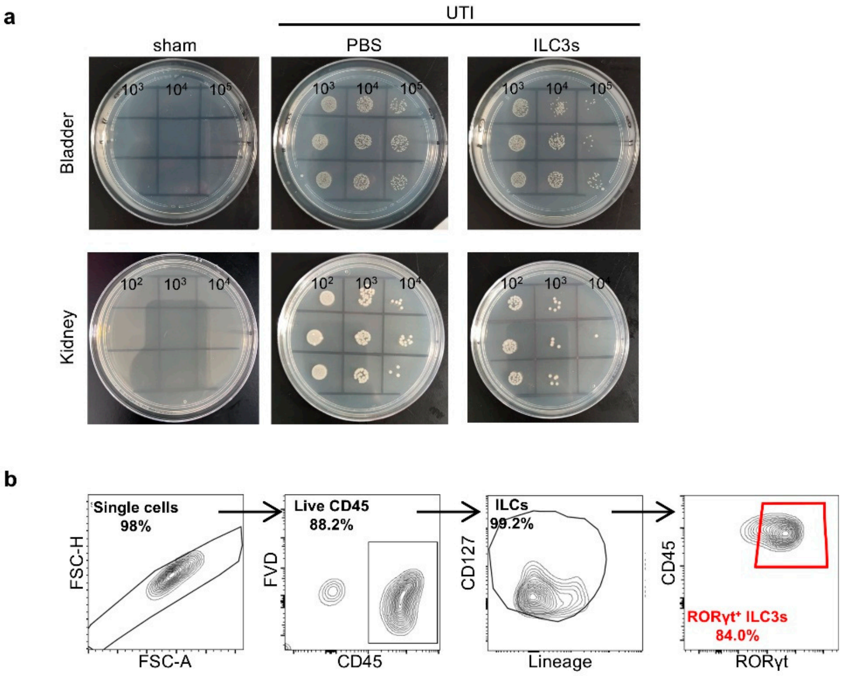

Figure S2. Representative images of bacterial load and purity of sorted ILC3s. (a) Representative images of bacterial growth on agar plates from bladder and kidney, corresponding to the quantitative data presented in Figures 2j,k. Each row in the images represents the colony formation resulting from inoculating serially diluted homogenates of bladder or kidney from an individual mouse onto agar plates. (b) Gating strategy used to identify purity of sorted ILC3s.

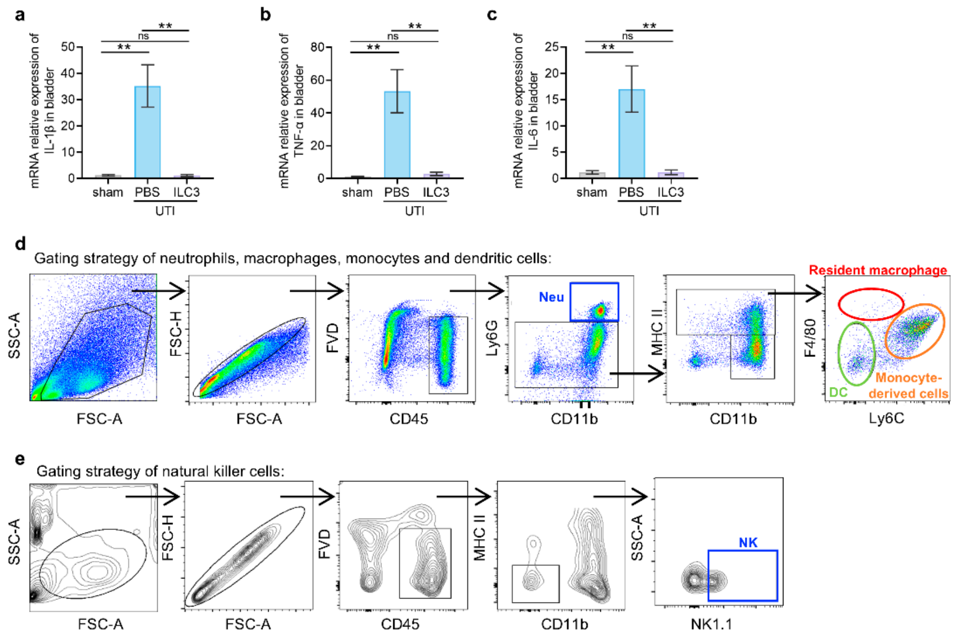

Figure S3. Assessment of Bladder Inflammatory Infiltration. (a-c) The mRNA expression of IL-1β, TNF-α and IL-6 in bladders was assessed by real-time qPCR. (d) Gating strategy used to identify neutrophils, resident-macrophages, monocyte-derived cells and DCs in bladder. (Neutrophils were defined as CD45+CD11b+Ly6G+ cells, resident macrophages were defined as CD45+CD11b+Ly6G-MHCII+Ly6C^{int}F4/80^{high} cells, monocyte-derived cells were defined as CD45+CD11b+Ly6G-MHCII+Ly6C^{high}F4/80^{int} cells, and DCs were defined as CD45+CD11b+Ly6G-MHCII+Ly6C-F4/80- cells.) (e) Gating strategy used to identify NK cells in bladder. NK cells were defined as CD45+CD11b-MHC II-NK1.1+ cells. Data are shown as mean ± SEM. One-way ANOVA test was performed. **p < 0.005; ns, not significant.

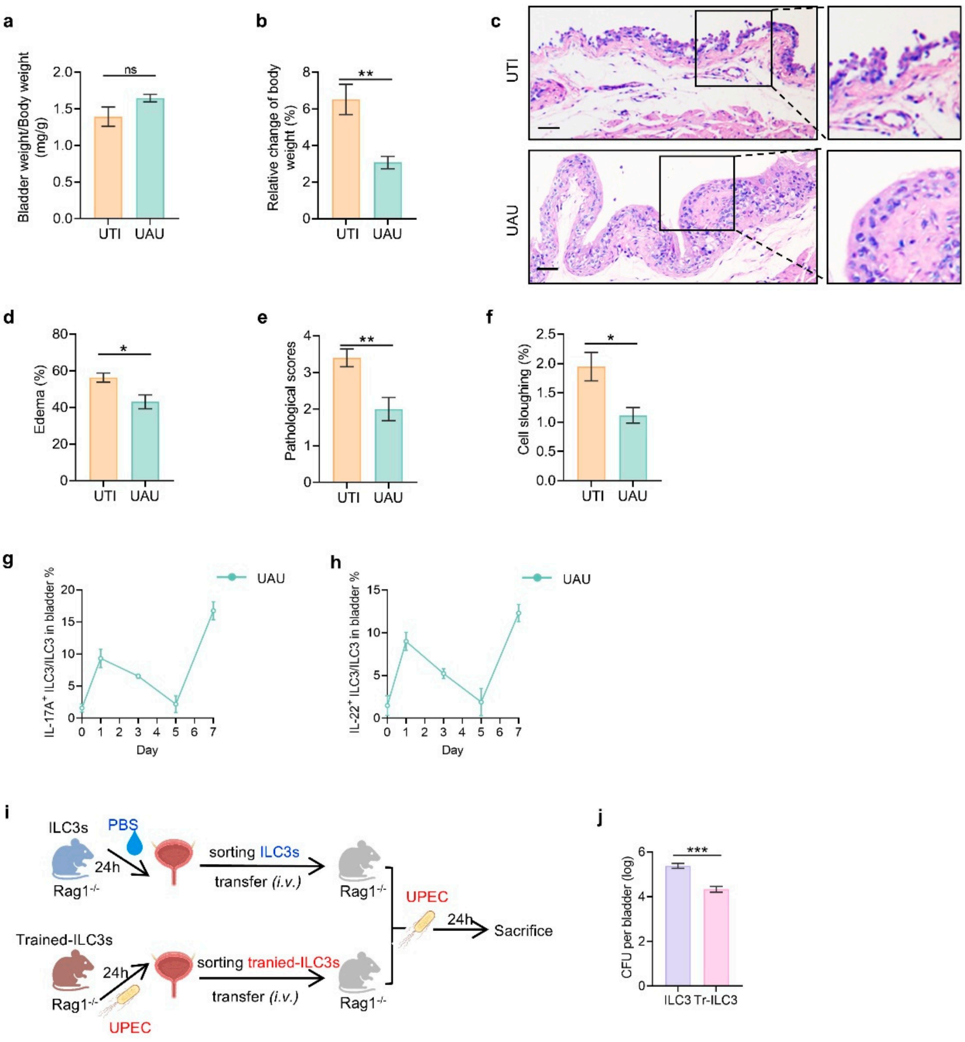

Figure S4. Trained ILC3s Confer Enhanced Protection During Secondary Bladder Infection. (a,b) The ratio of bladder weight to body weight (a) and relative change of body weight (b) of UTI and UAU mice were measured 24 hours post secondary infection. (c) Representative images of H&E-stained bladder sections. Scale bars=50μm. (d-f) The percentage of subepidermal lamina propria edema in the urothelium (c), pathological scores (d), and the percentage of epithelial cell sloughing (e) were analyzed based on H&E-stained bladder sections. Data are shown as mean ± SEM. Student's t-test was performed. $^{**}p < 0.005$; $^{*}p < 0.05$; ns, not significant. (g,h) IL-17A (g) and IL-22 (h) expression of ILC3 monitored over the infection timeline. (i) Schematic of experimental design for adoptive transfer of naïve or Tr-ILC3s in Rag1$^{-/-}$ mice. (j) Bladder bacterial burden of Rag1$^{-/-}$ mice adopting ILC3 or Tr-ILC3.

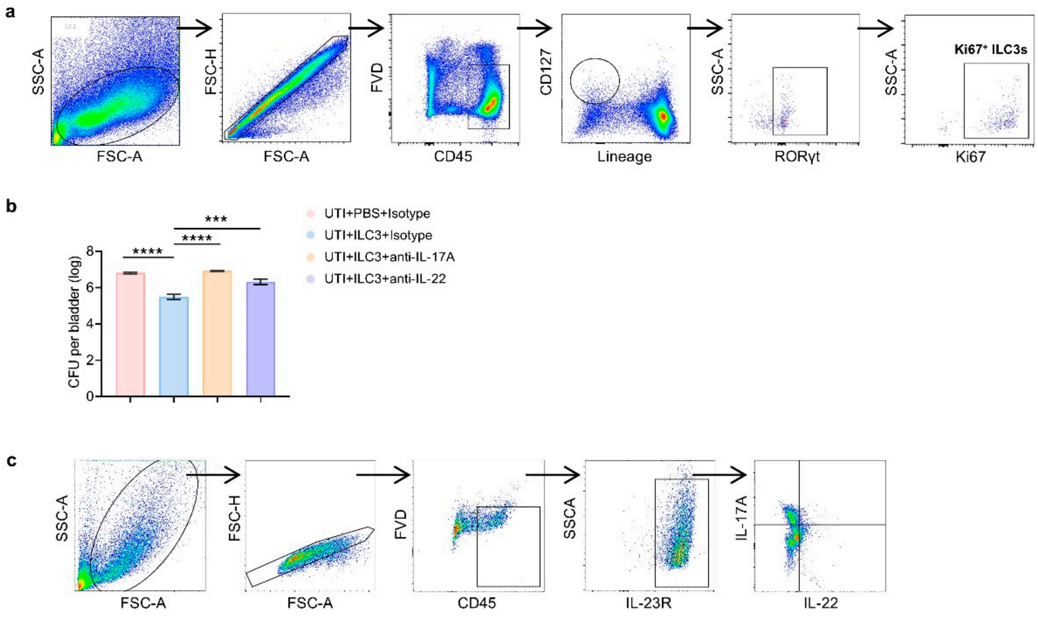

Figure S5. Evaluation of disease severity and bladder histopathology post-secondary infection. (a) Gating strategy used to identify Ki67+ ILC3s. (b) Bladder bacterial burden in UTI mice following transfer of PBS or ILC3s, with neutralizing antibodies against IL-17A, IL-22, or an isotype control. (c) Gating strategy used to identify IL-17A/IL-22 expression of MNK-3.

Table S1. The primer sequences of target genes.

Gene Name	Forward primer sequence (5′ to 3′)	Reverse primer sequence (3′ to 5′)
Gapdh	AGGTCGGTGTGAACGGATTTG	GGGGTCGTTGATGGCAACA
Il1b	GAAATGCCACCTTTTGACAGTG	TGGATGCTCTCATCAGGACAG
Il6	CTGCAAGAGACTTCCATCCAG	AGTGGTATAGACAGGTCTGTTGG
Tnfa	CCCTCACACTCAGATCATCTTCT	GCTACGACGTGGGCTACAG
Ifng	ATGAACGCTACACACTGCATC	CCATCCTTTTGCCAGTTCCTC
Il22	ATGAGTTTTTCCCTTATGGGGAC	GCTGGAAGTTGGACACCTCAA
Il17a	TGAGCTTCCCAGATCACAGA	TCCAGAAGGCCCTCAGACTA
Csf2	CTGCTCTTCTCCACGCTACTG	GAGACTCGCCGGTGTATCC

Reg3b	ACTCCCTGAAGAATATACCCTCC	CGCTATTGAGCACAGATACGAG
Reg3g	ATGCTTCCCCGTATAACCATCA	GGCCATATCTGCATCATACCAG
S100a8	AAATCACCATGCCCTCTACAAG	CCCACTTTTATCACCATCGCAA
S100a9	ATACTCTAGGAAGGAAGGACACC	TCCATGATGTCATTTATGAGGGC

Table S2. Clinical characteristics of healthy controls and UTI patients detected for renal ILC3s by immunofluorescence.

Group	Gender (M/F)	Age in years
Healthy controls	7/3	67.3±10.34
UTI patients	2/7	64.11±18.23

Data are presented as n or mean ± standard deviations. M: male; F: female.
